# Evidence for Alloimmune Sinusoidal Injury in *De Novo* Nodular Regenerative Hyperplasia After Liver Transplantation

**DOI:** 10.3389/ti.2023.11306

**Published:** 2023-07-26

**Authors:** Mylène Sebagh, Funda Yilmaz, Ilias Kounis, Faouzi Saliba, Cyrille Feray, Jean-Luc Taupin, Daniel Cherqui, Daniel Azoulay, Didier Samuel, Audrey Coilly, Antony-Jake Demetris, Desley Neil

**Affiliations:** ^1^ Laboratoire d’Anatomopathologie, AP-HP Hôpital Paul-Brousse, Villejuif, France; ^2^ Inserm, Unité 1193, Université Paris-Saclay, Villejuif, France; ^3^ Université Paris-Saclay, Villejuif, France; ^4^ Ege University Organ Transplantation Center, Department of Pathology, School of Medicine, Ege University, Bornova, Izmir, Türkiye; ^5^ Centre Hépato-Biliaire, AP-HP Hôpital Paul-Brousse, Villejuif, France; ^6^ Département d’Immunologie and d’Histocompatibilité, AP-HP Hôpital Saint-Louis, Paris, France; ^7^ Division of Transplantation, Medical Center, University of Pittsburgh, Pittsburgh, PA, United States; ^8^ Cellular Pathology, Queen Elizabeth Hospital, Birmingham, United Kingdom

**Keywords:** liver transplantation, nodular regenerative hyperplasia, Banff criteria, chronic antibody-mediated rejection, pathology, C4d

## Abstract

Posttransplant nodular regenerative hyperplasia (NRH) mostly remains unexplained. Microvascular injury due to antibody-mediated rejection (AMR) is suspected, but lack of donor specific antibody (DSA) testing makes it difficult to prove. Centered around a 1-year period of routine DSA testing, concomitant protocol, and indicated posttransplant liver biopsies (LB), recipients with NRH (*n* = 18) were compared with a matched control group (*n* = 36). All index, previous, and subsequent LB were reviewed. Both groups were similar in terms of demographics, timing of index LB, and DSA. In the index LB, the NRH group had higher sinusoidal C4d positivity (*p* = 0.029) and perisinusoidal fibrosis (*p* = 0.034), both independently associated with NRH (*p* = 0.038 and 0.050, respectively). Features of “possible” chronic AMR were detected in 28.5% of the NRH group without a known cause and 0% of the control group (*p* = 0.009). The NRH group had more preceding indicated LB with increased incidence of rejection and biliary obstruction pattern. In the follow-up histology, overall, sinusoidal and portal C4d positivity, sinusoidal microvasculitis, and perisinusoidal fibrosis were also higher (all *p* < 0.050). In conclusion, we provide evidence towards the hypothesis that some cases of posttransplant NRH are related to preceding active and persistent AMR. Large multicenter studies with protocol DSA testing are required to confirm.

## Graphical Abstract

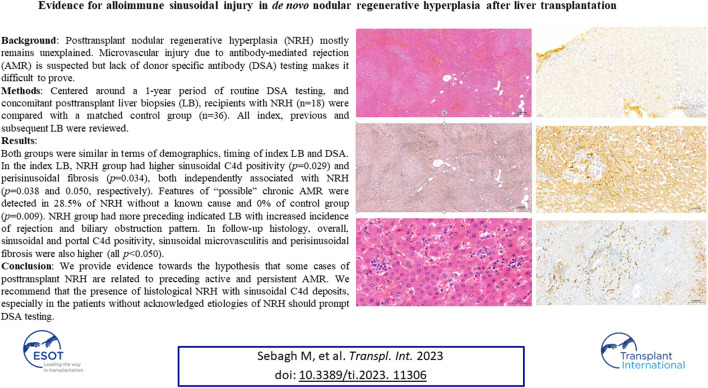

## Introduction

Nodular regenerative hyperplasia (NRH) is characterized by the diffuse transformation of the liver parenchyma into regenerative nodules with little-to-no perisinusoidal fibrosis [1]. In native livers, NRH is generally attributable to abnormalities in intrahepatic blood flow in small portal vein branches or hepatic venous drainage, and during the early stages of biliary tract disorders before more blatant cholangiopathic changes become obvious. NRH is associated with an ever-growing group of extrahepatic diseases and therapeutic agents including various immunological disorders, hematopoietic diseases, solid organ and bone marrow transplantation, and treatment with immunosuppressive or chemotherapeutic agents [2–4]. NRH can be asymptomatic and severe cases can show evidence of portal hypertension. Serum alkaline phosphatase and gamma glutamyl transpeptidase levels can be mildly elevated, but serum transaminase levels are usually normal [3].

The development of NRH following liver transplantation (LT) is not well documented, with only a few case reports [5–8] and three retrospective series in adult recipients [9–11]. NRH is seen with increasing frequency and with increased graft longevity [12, 13]. Most cases are asymptomatic, diagnosed on protocol liver biopsies (LB); however, some cases require retransplantation due to portal hypertension. Of the etiologies for NRH in native liver, azathioprine and vascular issues are higher up on the list of differential causes, however the number of unexplained cases remains high [11]. Porto-sinusoidal vascular disease (PSVD), which produces NRH is reported as an uncommon cause of recurrent disease after LT [14]. Chronic antibody-mediated rejection (cAMR), less well defined than acute antibody-mediated rejection (aAMR), is suspected by the Banff group to be one of the likely causes of NRH [15, 16]. Based on limited prior studies published before the era of donor specific antibodies (DSA), it is difficult to document this relationship and establish criteria for AMR-related NRH. Few centers do protocol LB and DSA testing. HLA DSA testing is haphazard, hepatologist-dependent, and non-HLA DSA is even less frequently tested. In the most recent series [11], based on a chart review without looking for histological clues to the potential etiology, no unifying risk factors were found, but the data pointed towards an immune-mediated process in the development of NRH.

The aim of this study was to get more concrete evidence for the relationship between AMR and the development of NRH, based on a detailed histological assessment for features of AMR from protocol and indicated LB taken during a 1-year window of protocol HLA DSA testing. The biopsies preceding and subsequent to the index LB were examined to look at sequential changes.

## Patients and Methods

### Study Population

In our center, DSA testing is not routinely conducted, except for patients on the waiting list for retransplantation or with unexplained graft dysfunction. For the purpose of the current study, case identification was restricted to 2014 as this was the sole period where DSA testing was performed systematically as part of a parallel study to know the incidence of DSA in the LT population. Patients were initially selected from the Pathology Department database using search criteria prospectively coded for a histological diagnosis of NRH made in 2014. Biopsy-proven NRH patients from this period, with synchronous DSA testing, were included (Supplementary Figure S1). The control group was formed by matching each NRH patient with two non-NRH patients who were biopsied in the nearest timeframe (just before and just after a given NRH patient). The study was conducted in accordance with the Declaration of Helsinki and French law for medical research. Free and informed consent was obtained for all the patients included.

Regular assessment includes a clinical, biochemical, and serological screening and calcineurin inhibitors doses at least every 6 months. LB were either “indicated” due to clinical and/or biochemical reasons or part of the systematic posttransplantation “protocol” at 1, 2, 5, 10, 15, and 20 years, independently of the donor and recipient HLA typing. Regarding immunosuppression, induction therapy such as IL-2 receptor antibodies (basiliximab) and anti-thymocyte globulin was used in patients with kidney dysfunction and those at higher immunological risk (retransplantation, immune-mediated liver disease, multiorgan recipient, highly sensitized) compared with essentially all other recipients who are considered lower immunological risk. A maintenance immunosuppression regimen is usually based on steroids (tapered and stopped between 3 and 6 months after LT), calcineurin inhibitors (tacrolimus or cyclosporine, mainly in HCV positive patients) and mycophenolate mofetil.

### Pathology Studies

**FIGURE 1:**
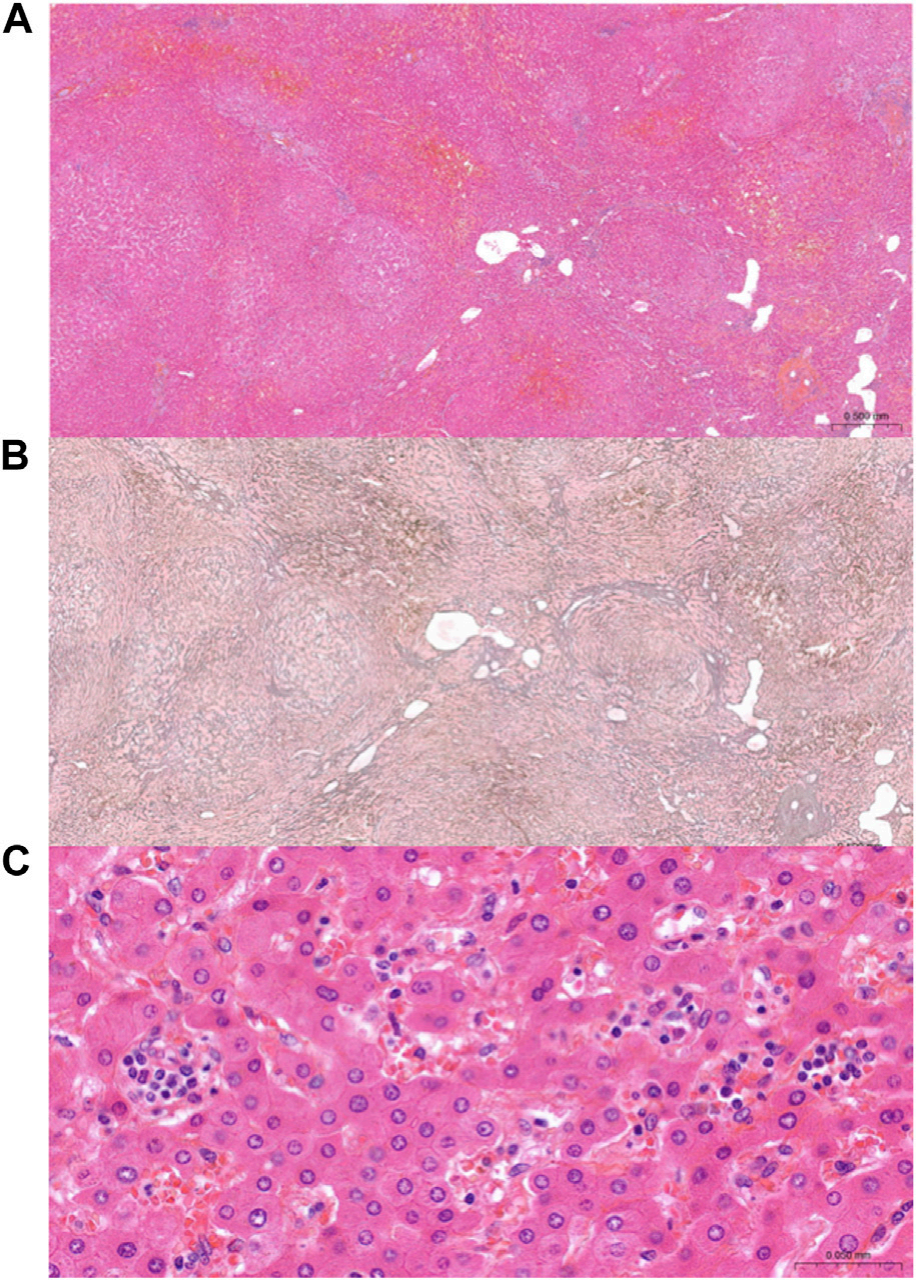
Representative images of NRH and “sinusoidal microvasculitis.” Alternating widened and atrophic hepatic plates in a nodular architecture, consistent with NRH on Hematoxylin eosin staining **(A)** and Gordon Sweet’s silver staining **(B)**. At high magnification, so-called “sinusoidal microvasculitis” **(C)** by analogy with portal capillaritis, defined by the presence of marginated monocytes/macrophages within dilated sinusoids.

The specimens were routinely paraffin-embedded and stained with hematoxylin-eosin-safran and Picrosirius. Index, previous, and subsequent LB and/or explants were reviewed by two experienced liver transplant pathologists (FY and MS) blinded to the clinical status. Disagreements between the two readers were minor and resolved by consensus meeting. The diagnosis of NRH was based on diffuse transformation of the liver parenchyma (confirmed by Gordon Sweet’s silver staining for reticulin, Figure 1) into regenerative nodules. Particular attention has been paid to histological features of AMR, as described elsewhere [15]. In short, histopathological pattern of injury consistent with aAMR mainly includes portal changes (i.e., microvascular endothelial cell hypertrophy, capillary and inlet venule dilatation, microvasculitis, edema, and ductular reaction). Among them, microvasculitis is the histopathological “signature” of aAMR. It can also affect the sinusoids (Table 4 of the Banff document [15]). Histopathological pattern of injury consistent with cAMR includes both unexplained and mononuclear portal and/or perivenular inflammation with interface and/or perivenular necro-inflammatory activity, and portal/periportal, sinusoidal and/or perivenular fibrosis. Portal microvasculitis is potentially observed in cAMR.

Here, the presence of monocyte/macrophage clusters of more than 5 cells within dilated sinusoids in most inflamed areas randomly in the lobules was named as “sinusoidal microvasculitis.”

### Immunostaining

**FIGURE 2:**
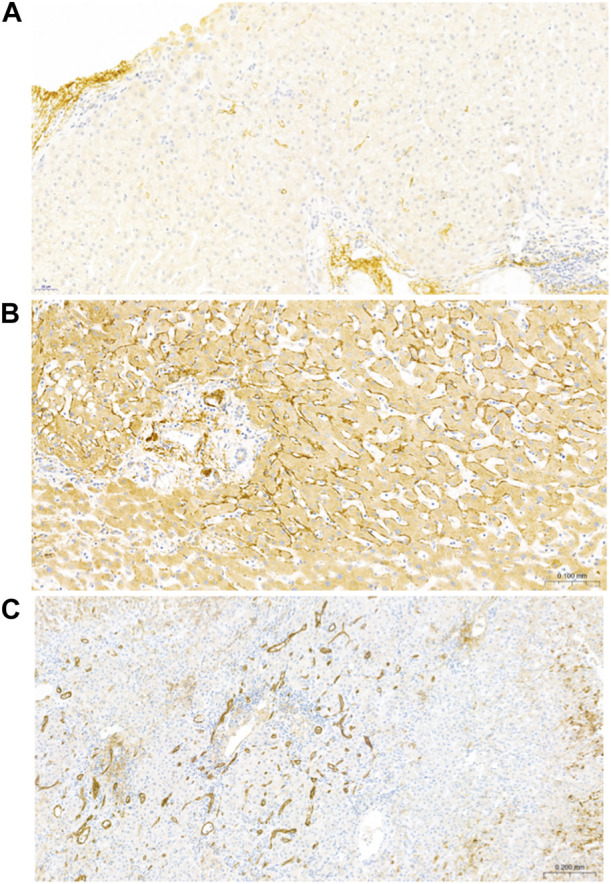
Representative images of C4d in patients with NRH. **(A)** An example of minimal (<10% staining) C4d positivity in an index LB with NRH. C4d positivity is observed within the sinusoidal microvasculature. **(B)** Focal (10%–50% staining) C4d positivity within the sinusoidal microvasculature in an explant with NRH. C4d deposition on the elastic fibers of arterioles was regarded as a positive internal control. **(C)** Diffuse (>50% staining) C4d positivity in an explant with NRH. C4d positivity is observed within the portal venules, capillaries and inlet venules, and sinusoids.

Immunostaining for C4d (rabbit monoclonal A24-T Biotech, Kosice, Slovakia) was evaluated in the compartments defined by the Banff group in portal veins and portal capillaries [15], but also in the centrilobular veins and sinusoidal endothelial cells. C4d immunostaining was scored as negative (score 0), minimal (<10%, score 1), focal (10%–50%, score 2) and diffuse (>50%, score 3) of structures in the index LB and last follow up histology in both groups (Figure 2).

In the NRH group, a panel (CD3, CD20, CD4, CD8, and CD68) was performed for immunotyping the sinusoidal infiltrate in the cases with sinusoidal microvasculitis. Changes in liver sinusoidal endothelial cell (LSEC) and hepatic stellate cell (HSC) phenotype were studied by comparing similarly-sized portal tracts, central veins, and sinusoids in the index LB versus last follow up histology stained for CD34 (mouse monoclonal, QBE-10, DAKO) and α-smooth muscle actin (α-SMA, mouse monoclonal, 1A4; DAKO). The expression of major histocompatibility complex (MHC) class II antigen (mouse monoclonal CR3/43; DAKO (MO775, Carpinteria, CA), the putative target of Class II DSA, was assessed by compartment.

### Assays for Anti-HLA Antibodies

Donors were typed for the HLA system using commercially available serological methods (One Lambda, Inc., Canoga Park, CA). Loci A, B, DP, DQ, and DR were typed. Blood samples were harvested from the recipients at the time and after the index LB. Recipient anti-HLA antibodies were retrospectively analyzed by Luminex with the LABScreen single antigen class I and single antigen class II beads (LS1A04 and LS2A01, respectively; One Lambda, Inc.), after neutralization of the complement interference phenomenon using ethylene diamine tetraacetic acid before treatment of the serum for all the samples that were found positive using the screening assay (LSM12; One Lambda, Inc.). Normalized mean fluorescence intensity (MFI) values of DSA were reported, using the baseline formula from the Fusion^®^ software. The specificities for both class I and II HLA antibodies were considered significant for MFI >1,000 in accordance with the cutoff values used in LT [17].

### Statistical Analysis

NRH and control patients were compared in terms of demographic data, histopathological features, immunostaining for C4d, and DSA. Student’s t-test was used for continuous variables, whereas the chi-squared test or Fisher exact test (for small numbers) was applied to analyze categorical variables. The variables were first considered under univariate analysis. Those with *p* < 0.15 (because of the small sample size) were then tested by logistic regression analysis. A *p*-value of <0.05 was considered to be significant.

## Results

### Patients

During the study period, a total of 356 LB were performed in 329 liver transplant patients (Supplementary Figure S1). Twenty-three (6.4%) patients were diagnosed with NRH in 23 (6.9%) LB. Among them, five patients were excluded due to lack of synchronous DSA testing. The study included 18 NRH patients and 36 matched controls. Table 1 gives patients’ characteristics. There were 14 males and 4 females, with a mean age of 50.7 + 11.6 years at the time of the initial LT. None of the patients were infected by HIV. The majority of patients were transplanted for cirrhosis. None were transplanted for NRH. None of the patients had systemic diseases, prothrombotic status, or hematological disorders. One patient concomitantly underwent kidney transplantation for chronic interstitial nephropathy and one underwent heart transplantation 4 years after LT for amyloidosis-related cardiac insufficiency.

**TABLE 1:** Characteristics of the liver transplant patients from NRH and control groups.

	NRH group (*n* = 18)	Control group (*n* = 36)	*P* ^#^
Gender (M/F)	14/4	20/16	0.142
Mean age at initial transplantation	50.7 ± 11.6	51.4 ± 11.5	0.690
Native disease			
Alcoholic cirrhosis	2 (11.1)	8 (22.2)	0.466
HCV-cirrhosis	4 (22.2)	12 (33.3)	0.532
HBV-cirrhosis	3 (16.7)	8 (22.2)	0.733
NASH-cirrhosis	1 (5.6)	0 (0.0)	
Fulminant hepatitis	1 (5.6)	3 (8.3)	
Amyloidotic neuropathy	3 (16.7)	1 (2.8)	
Primary biliary cholangitis	0	3	
Primary sclerosing cholangitis	1	1	
Biliary atresia	2	0	
Tyrosinemia	1	0	
Concomitant HCC	5 (27.8)	7 (19.4)	0.506
Chemotherapy for prevention of HCC recurrence	2/5	0/7	
Immunosuppression			
Induction therapy	3 (16.7)	8 (22.2)	0.733
Maintenance regimen			
Tacrolimus	14 (77.8)	24 (66.7)	0.532
Cyclosporine	3 (16.7)	10 (27.8)	0.506
Everolimus	1 (5.6)	2 (5.6)	
Mycofenolate mofetil	16 (88.9)	24 (66.7)	0.105
Azathioprine	1 (5.6)	0 (0.0)	
Index LB			
Mean post-transplant time (years)	6.2 ± 6.3	6.4 ± 6.0	0.640
Nature of the index LB			
Indicated LB	4 (22.2)	3 (8.3)	0.182
Routine LB	14 (77.8)	33 (91.7)	0.204
Abnormal LFTs at the time of the index LB regardless of its nature	11 (61.1)	10 (27.8)	**0.036**
Cholestasis	7	4	
AST and/or ALT elevation	0	2	
Both	4	4	
Surgical complications	3	5	1.000
Biliary stenosis	1 (5.6)	2 (5.6)	
Portal vein thrombosis	2 (11.1)	0 (0.0)	
Arterial stenosis	0 (0.0)	2 (5.6)	
Arteriolo-venous fistula	0 (0.0)	1 (2.8)	
Follow up			
Available histological follow-up	14 (77.8)	25 (69.4)	0.748
Follow-up course			
Death	1 (5.6)^a^	1 (2.8)^b^	1.000
Retransplantation	5 (27.8)	0 (0.0)	**0.003**

Values presented as n (%), Liver biopsies (LB), HCC, hepatocellular carcinoma; LB, liver biopsy; LFTs, Liver Function Tests.

^#^ *p* ≤ 0.05 was considered statistically significant (in bold).

^a^ Death due to sepsis.

^b^ Death due to colon carcinoma.

Regarding immunosuppression, induction therapy was used in eight patients. Fourteen (77%) patients were on maintenance immunosuppression with tacrolimus, three (16%) with cyclosporine and one (5%) with everolimus. Sixteen (89%) received mycophenolate mofetil (MMF) and one (n°14) received azathioprine since LT (10 years ago). There was no change in immunosuppression following the diagnosis of NRH.

Both groups were similar in terms of demographics such as sex, mean age at the time of LT, native disease, induction therapy (8/36 versus 3/18) and maintenance immunosuppression, which was mainly based on tacrolimus (24/36 versus 14/18) and MMF (24/36 versus 16/18). The two groups did not differ in terms of mean posttransplant timing of index LB (6.4 ± 6.0 versus 6.2 ± 6.3 years), and indication for index LB (clinically indicated in 3/36 versus 4/18, or protocol LB in 33/36 versus 14/18). Regardless of the indication for index LB, abnormal LFT were significantly less frequent in the control group (*p* = 0.036).

Graft loss was higher in the NRH group (5/18 versus 0/36, *p* = 0.003). Patients were retransplanted at a mean time of 8.1 ± 5.9 years after LT and at a mean interval of 2.1 ± 0.9 years after the diagnosis of NRH. The reason for retransplantation was related to NRH complicated by portal hypertension (refractory ascites in 3, variceal bleeding in 2). Patient survival was similar in both groups.

### Pathological Results

#### Previous LB in Both Groups

Biopsies prior to the index LB were performed in 15 NRH patients and 26 control patients who underwent 47 and 76 LB, respectively. The mean and median number of previous LB were 2.3 ± 2 and 2 (range: 0–8) in the NRH group, and 2 ± 2 and 2 (range: 0–6) in the control group. The number of indicated LB was significantly higher in the NRH group (32/47 versus 27/76, *p* = 0.001) (Table 2).

**TABLE 2:** Comparison of main histopathological features, C4d immunostaining, and DSA between NRH and control groups.

	n (%)		
	NRH *n* = 18	Controls *n* = 36	P^#^
Previous liver biopsies (LB)	47	76	
Median (range)/Mean number of LB	2 (0–8)/2.3 + 2	2 (0–6)/2 ± 2	1.000
Number of patients with at least one previous LB	15 (83.3)	26 (72.2)	0.506
Number of protocol/indicated LB	15/32	49/27	**0.001**
Rejection	7	4	**0.029**
TCMR	4	3	
Chronic ductopenic rejection	1	1	
Plasma cell rich rejection	1	0	
aAMR	1	0	
Biliary obstruction pattern	3	0	**0.033**
Sinusoidal microvasculitis	4	5	0.461
Index liver biopsies			
TCMR	0 (0.0)	2 (5.6)	0.547
Chronic ductopenic rejection	2 (11.1)	4 (11.1)	1.000
C4d positivity	8 (44.4)	8 (22.2)	0.119
Portal compartment	3 (16.7)	5 (13.8)	1.000
Sinusoidal compartment	7 (38.9)	4 (11.1)	**0.029**
Centrilobular vein	0 (0.0)	2 (5.6)	1.000
Sinusoidal microvasculitis	6 (33.3)	5 (13.9)	0.150
Perisinusoidal fibrosis	6 (33.3)	5 (13.9)	0.150
Perisinusoidal fibrosis unrelated to NASH	5 (27.8)	2 (5.6)	**0.034**
Last histological follow up	14 (77.8)	25 (69.4)	0.748
TCMR	1 (7.1)	0 (0.0)	0.358
Chronic ductopenic rejection	2 (14.2)	2 (8.0)	0.61
C4d positivity	10 (71.4)	1 (4.0)	**<0.001**
Portal compartment	5 (35.7)	1 (4.0)	**0.021**
Sinusoidal compartment	10 (71.4)	0 (0.0)	**<0.001**
Centrilobular vein	0 (0.0)	0 (0.0)	1.000
Sinusoidal microvasculitis	7 (50.0)	3 (12.0)	**0.019**
Perisinusoidal fibrosis	5 (35.7)	4 (16%)	0.238
Perisinusoidal fibrosis unrelated to NASH	3 (21.4)	0 (0.0)	**0.039**
DSA at the time of index liver biopsies			
Positive DSA	6 (33.3)	12 (33.3)	1.000
More than one DSA	4 (22.2)	2 (5.6)	0.087
Class II DSA	5 (27.8)	11 (30.6)	1.000
High MFI (>1,000) class II DSA	5 (27.8)	7 (19.4)	0.506
Class I DSA	3 (16.7)	1 (2.8)	0.102
High MFI (>1,000) class I DSA	1 (5.6)	1 (2.8)	1.000
Chronic AMR	4 (22.2)	0	**0.009**

TCMR, T cell-mediated rejection; AMR, antibody-mediated rejection; DSA, donor specific antibodies; MFI, mean fluorescence intensity; NRH, nodular regenerative hyperplasia.

^#^ *p* ≤ 0.05 was considered statistically significant (in bold).

Among overall previous LB to index ones, features consistent with aAMR were present in one patient of the NRH group and in none of the control group. T cell-mediated rejection (TCMR) was more common in the NRH group (22% versus 8%) without reaching significance. Ductopenic rejection was observed in one NRH patient and one control patient. The pattern of biliary obstruction was significantly more common in the NRH group (*p* = 0.033). This was observed only in the NRH group and in the absence of abnormalities of the biliary imaging. The presence of sinusoidal microvasculitis was similar between both groups.

#### NRH Group

On the index LB, NRH was confirmed in all of them. Ductopenic rejection was concomitant to NRH in two and TCMR in none. No portal capillaritis was observed. Sinusoidal microvasculitis was observed in six (33%) LB. Perisinusoidal fibrosis was observed in six (33%) LB, of whom five were unrelated to NASH. No microthrombotic changes were observed in sinusoids or portal venules. Immunostaining for C4d was positive in eight (44.4%) LB. Positivity was observed only in portal venules in one (minimal), only in sinusoids alone in five (minimal in four and focal in one, respectively) and in both in two LB (focal in one and diffuse in one, respectively).

**FIGURE 3:**
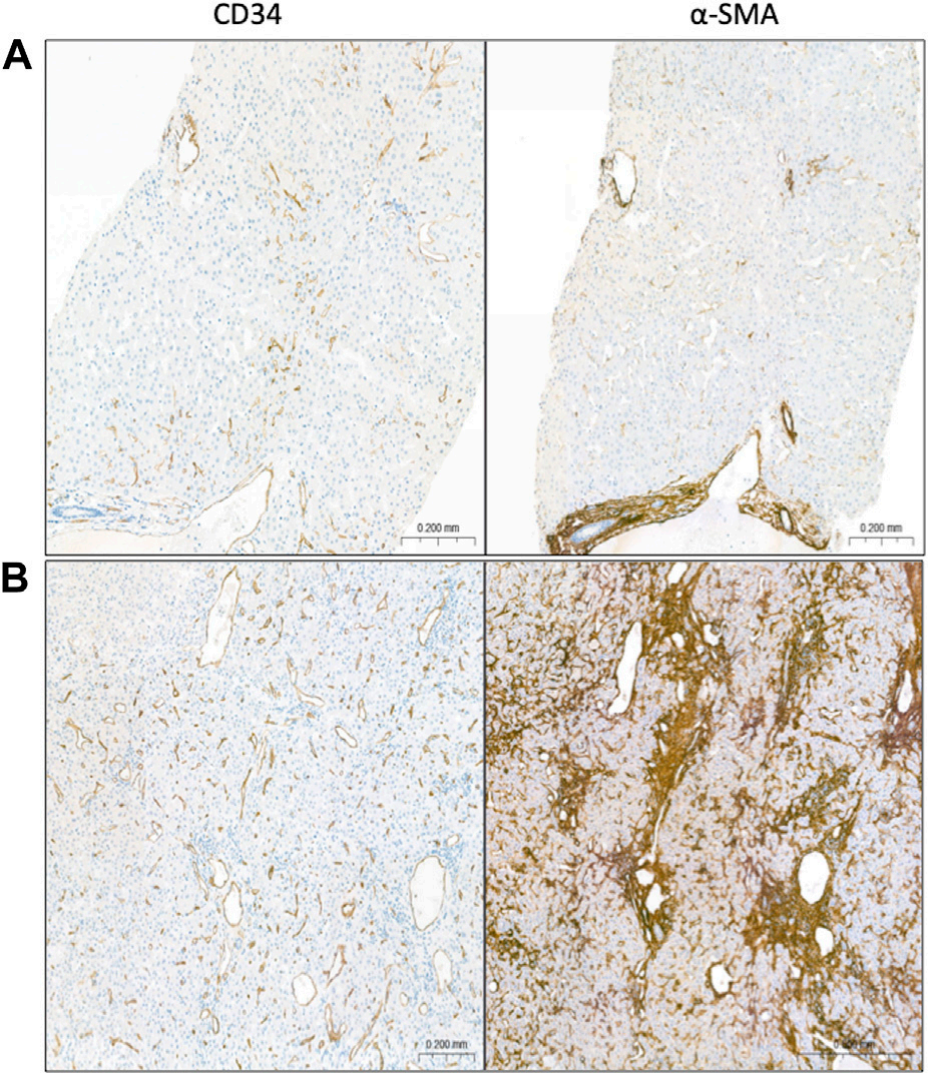
Shift toward a pathogenic phenotype in HSC and LSEC over time. By comparing similarly-sized portal tracts, central veins, and sinusoids in the index LB **(A)** versus last follow up histology **(B)**, there was an increase in CD34 expression in peri-portal, sinusoidal, or peri-venular regions, and in α-SMA expression diffusely.

**FIGURE 4:**
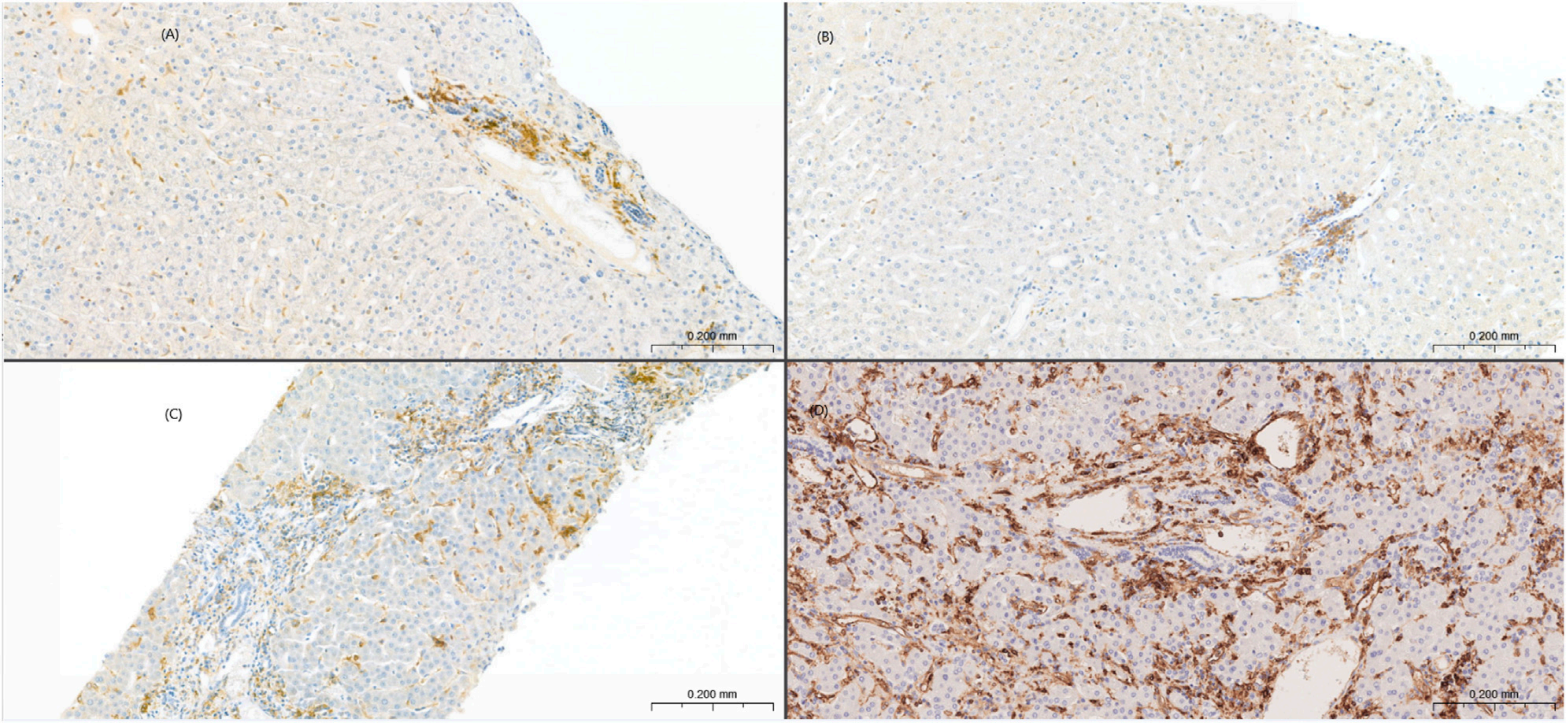
Sinusoidal MHC class II overexpression in NRH. Low MHC class II expression limited focally on sinusoidal endothelium in the index **(A)** and last follow up LB **(B)** from a control patient. Sinusoidal MHC class II overexpression in the index **(C)** and last follow up LB **(D)** from a NRH patient. Portal-based dendritic cells served as internal positive controls.

On last follow up histology available in 14 cases including 9 LB and 5 explants, we observed persistence of NRH. No microthrombotic changes were observed in sinusoids. Two explants showed portal venopathy. The global incidence of C4d deposits and sinusoidal microvasculitis (71.4% and 50%, respectively) increased as compared with the index LB (44% and 33%, respectively) without reaching significance. For a given patient, C4d deposits, either in portal tracts or sinusoidal, increased in 50% of patients in the follow-up LB after the index LB. The sinusoidal infiltrate contained abundant lymphocytes of predominantly CD3/CD8 phenotype in all cases with sinusoidal microvasculitis. We detected a shift toward a pathogenic phenotype in HSC and LSEC (Figure 3): Over time, there was an increase in CD34 expression in peri-portal, sinusoidal, or peri-venular regions, and in α-SMA expression diffusely. MHC Class II staining increased dramatically, predominantly in the sinusoidal compartment (Figure 4).

#### Control Group

On the index LB, the main pathological diagnoses were as follows: normal in six, steatosis/non-alcoholic steatohepatitis in nine, ductopenic rejection in four, TCMR in three, sinusoidal congestion in four, chronic hepatitis in nine, and biliary obstruction in one. No NRH changes were observed. No portal capillaritis was observed. Sinusoidal microvasculitis was observed in five (14%) LB. Perisinusoidal fibrosis was observed in five (14%) LB, of whom two were unrelated to NASH. Immunostaining for C4d was positive in eight (22%) LB. Positivity was observed only in sinusoids in 3 (minimal in 1 and focal in 2, respectively), only in portal venules in 2 (minimal), in both portal venules and sinusoids in 1 (minimal), and in both portal and centrilobular venules in 2 LB (minimal).

Last histology showed no NRH changes and no significant fibrosis, and only one (1/25) had minimal portal C4d deposit.

#### Comparison of Main Histological and Immunohistochemical C4d Features of Index LB and Subsequent Histology Between Groups

There is a trend towards increased sinusoidal microvasculitis in the NRH group in the index LB (33% versus 14%) and previous LB (27% versus 19%), however this does not reach statistical significance (using univariate analysis). Sinusoidal C4d positivity and perisinusoidal fibrosis in index LB were higher in NRH group (*p* = 0.029 and 0.034, respectively). Under multivariate analysis, sinusoidal C4d deposits and perisinusoidal fibrosis were independently associated with NRH (*p* = 0.038 and 0.050, respectively).

In follow-up histology, higher sinusoidal microvasculitis, perisinusoidal fibrosis, and overall, sinusoidal and portal C4d positivity (all *p* < 0.050) were observed in the NRH group. Multivariate analysis was not possible because the number of patients with follow up histology was not sufficient.

### DSA at the Time of the Index LB

DSA were present in six (33%) patients of the NRH group in whom four had more than one DSA type (Table 2). Five patients exhibited class II DSA in significant MFI in all of them. Three patients had class I DSA in significant MFI in one and approaching significance in two of them. Two patients exhibited both class I and II DSA. Twelve (33%) patients of the control group had positive DSA in whom two had more than one DSA type. One patient had significant class I DSA. Eleven patients had class II DSA, in significant MFI in seven of them. There was no difference between the groups in terms of DSA (presence, number, class, and level of MFI).

### Diagnosis of cAMR

No patient from the control group could be classified as cAMR as they did not meet the histopathological features at index and follow-up.

In the NRH group, four patients (Supplementary Table S1, n°1, 3, 7, 11) with mainly sinusoidal and/or portal vascular C4d positivity, portal and/or perisinusoidal fibrosis in index LB and DSA positivity were classified as cAMR. Three of them were retransplanted. Explant livers showed progression of the histopathological features detected in the index LB, especially perisinusoidal fibrosis and sinusoidal microvasculitis. DSA in terms of type and level of MFI were comparable to those present at the time of the index LB. The fourth patient developed ascites and had no follow-up biopsy and no DSA testing. In these patients, no other causes to explain clinical and morphological findings and acknowledged etiologies of NRH were found. These four patients represented 22.2% (4/18) of all the NRH patients and 28.5% (4/14) of the NRH patients without acknowledged etiologies of NRH.

Three additional patients with NRH (n°2, 4, 15) had the histopathological criteria and C4d immunostaining consistent with cAMR in follow-up, but DSA were not tested in one patient (n°15) and remained negative in the two retransplanted patients (n°2, 4). In these two latter patients, portal vein thrombosis has been discovered after the diagnosis of NRH on the index LB in one (pt n°2), and before the index LB indicated for ascites in the other. Both explants found portal vein thrombosis and portal venopathy.

## Discussion

Our study is the first that attempts to investigate the relationship between posttransplant NRH and AMR. Despite inconsistent routine DSA testing overall, thanks to a 1-year period of routine DSA testing together with a long-standing system of protocol LB, we were able to look at a subset of our liver transplant patients with NRH. In comparing our cohort of posttransplant NRH to the control group, we found that the patients who develop NRH had more preceding indicated LB with an increased incidence of rejection diagnosis, including TCMR and features suspicious for AMR, or biliary features which are also a recognized feature of AMR, after exclusion of biliary obstruction. At the time of the index LB, the diagnosis of cAMR could be made in a subset of our NRH patients according to the current 2016 Banff criteria [15]. In addition to these, currently classifiable as cAMR, we identified an association with a form of progressive antibody-mediated sinusoidal injury consisting of persistent sinusoidal microvasculitis and sinusoidal C4d staining, evolving from the previous LB to the index LB and follow-up histology. These findings argue for the addition of NRH to the features of cAMR and should prompt DSA testing, especially in the absence of acknowledged etiologies of NRH.

Due to the short time period of routine DSA testing, the present study has several limitations, including its retrospective design, small sample size, and the inability to test some antibodies such as anti-endothelial antibodies at that time. The strengths of our study include a single-center experience, uniform diagnostic methods, and attentive post-LT care including performance of protocol LB.

As expected, the majority of NRH cases had no known associated risk factor for the development of NRH (14/18), with some requiring retransplantation for the consequences of non-cirrhotic portal hypertension/PSVD. Graft loss/retransplantation was higher (28%) in the NRH group than in the control group (0%) in whom no patient developed PSVD or cirrhosis. A significantly higher sinusoidal microvasculitis in follow up histology, and sinusoidal C4d accumulation and perisinusoidal fibrosis in the index LB and follow-up histology, were observed in the NRH group. First, a sinusoidal lymphocyte infiltrate has not been reported in NRH after LT but the case reports and the series did not provide sufficient histological data to be certain that this is the case. A sinusoidal microvasculitis has rarely been described in native livers with NRH either in the non-transplant setting or after transplantation of organs other than the liver. Ziol et al [18] described intrasinusoidal infiltrate composed of cytotoxic CD8+T-lymphocytes in 32% of patients with NRH. The T-cells were located near atrophic liver cell plates and adjacent to endothelial cells exhibiting evidence of apoptosis. The authors suggested the contribution of T-lymphocyte cytotoxicity against endothelial cells as a pathophysiologic mechanism in NRH with intrasinusoidal infiltrate as well as in NRH without intrasinusoidal infiltrates since the previous repeated LB demonstrated lymphocyte infiltration that decreased up to its complete disappearance. In contrast, in our study, higher sinusoidal microvasculitis was observed in the NRH group but only on the last follow up histology, and not prior to the development of the NRH. The late lymphocyte recruitment argues that it did not cause NRH. We ruled out the potential confounding factors such as recurrent disease, adverse drug reactions, and severe TCMR [19, 20]. We did not identify sinusoidal microthrombi in any case of NRH with or without infiltrate, although we would postulate that these have occurred previously at a time when a biopsy was not taken or that they were so subtle that they were not detected by standard staining [21]. Microthrombi have been reported as a result of endothelial injury/activation related to aAMR in liver graft [22, 23], analogous to a thrombotic microangiopathy seen as part of AMR in renal biopsies [24]. In native livers with PSVD, portal venopathy (identified in 2 of our NRH patients) is thought to result from previous microthrombotic events [25], which are attributed to recurrence in patients transplanted with common variable immunodeficiency for this indication [14].

Second, higher sinusoidal C4d deposits on index and last histology were observed in the NRH group. There is no data of C4d accumulation in native livers with NRH, as C4d immunostaining is not performed in these cases. It can be argued that sinusoidal C4d deposits can be “nonspecific” due to C4d binding to collagen around diseased sinusoids because of increased perisinusoidal fibrosis. We ruled out this hypothesis: Sinusoidal C4d deposits and perisinusoidal fibrosis were independently associated with NRH under multivariate analysis. In addition, we demonstrated MHC class II overexpression in sinusoids while native and graft liver display low MHC class II expression, limited predominantly to occasional portal capillaries and focally on sinusoidal endothelium as previously reported [26–29]. It is of note that, in the eight NRH patients and eight controls with C4d positivity, DSA was negative in four and in two, respectively. The principal targets of the humoral immune response are the highly polymorphic HLA antigens, but studies have also implicated antibodies directed against non-HLA autoantigens such as angiotensin type 1 receptor, perlecan, and collagen in the process of AMR [30, 31]. Unfortunately, none of our patients had available data regarding non-HLA antibodies to address this question.

Third, perisinusoidal fibrosis is not specific to NRH after LT, which can occur in the late stages of NRH in native livers, irrespective of the cause. The abnormal CD34 expression of LSEC reflects capillarization, lack of fenestration, and formation of an organized basement membrane, which are permissive for HSC activation, related-α-SMA positivity, and fibrosis [27, 28]. Irrespective of the etiologies, initial endothelial injury promotes the phenotypic changes in LSEC and HSC. The MHC class II overexpression could reflect an injury of immune-mediated nature.

The features of cAMR are described as low-grade chronic inflammation, progressive fibrosis, and microvascular C4d deposition in patients with (near) normal LFT and DSA positivity [15, 16, 32]. The denomination into «probable» and «possible» cAMR depends on the C4d score. From these actual 2016 Banff criteria, it is of note that: 1) the possible category: “DSA not available, equivocal, or negative,” present for the classification of aAMR is not defined for cAMR. It is the reason for which our NRH patients with other criteria for cAMR in follow-up but negative DSA in two and non tested-DSA in one were finally not classified as cAMR. In the two retransplanted patients, explants additionally showed portal venopathy, this feature being a part of AMR but also being due to the portal vein thrombosis; 2) C4d deposits are located into portal tracts. In a multicenter study [33], sinusoidal C4d deposits were rare and difficult to identify. One team twice reported sinusoidal C4d deposits as an indication of antibody-mediated response in liver allografts [34, 35]; 3) regarding progressive fibrosis, atypical fibrosis patterns have emerged including perisinusoidal and perivenular fibrosis [15, 36]; 4) low-grade chronic inflammation affects portal tracts and/or perivenular areas and “portal capillaritis” is potentially observed. Sinusoidal microvasculitis may be the morphological equivalent of the portal capillaritis; 5) the Banff group admitted that cAMR suffers from a lack of specific/typical features, and additionally suspected a spectrum of liver allograft injuries including non-inflammatory fibrosis, low-grade inflammation, biliary strictures, v-lesion, and NRH as histopathological features of cAMR [15, 29, 36, 37]. It is not clear if these injuries should be associated with all the established Banff criteria or whether their presence alone is sufficient for a diagnosis of AMR. Irrespective of the above caveats, by strictly applying the actual 2016 Banff criteria, four NRH patients (22.2% of all NRH patients and 28.5% of those where other likely causes of NRH were excluded) could be classified as “possible” cAMR, the most striking histological features were within the sinusoids: sinusoidal microvasculitis, sinusoidal C4d deposits, and perisinusoidal fibrosis.

The following question is raised: “is this just a co-incidence or is there a direct and causal relationship between AMR and NRH?” The comparison between both groups showed at least a significant association between both conditions. We believe this is related to AMR/DSA and not a non-specific response to circulating HLA antibodies, as there is no evidence that circulating HLA antibodies—when not donor specific (e.g., transplant of a different organ who develop antibody or sensitization following transfusion)—are linked to the development of NRH. For the development of AMR, the “second-hit” hypothesis has been proposed, summarized in Figure 1 from the review by Kim et al. [32] as follows: Injury in the liver allograft upregulates class II expression that facilitates class II DSA binding. Complement fixing antibodies may activate complement. Antibodies with Fc binding receptors may facilitate antibody dependent cellular cytotoxicity explaining the presence of sinusoidal lymphocytes. This demonstrates a sinusoidal localization of each step, supporting the sinusoidal and architectural changes, just as we observed (i.e., C4d deposits, MHC class II overexpression and microvasculitis). This also highlights a dynamic phenomenon: Here, follow up histology versus index LB showed increased sinusoidal and portal C4d deposits, and late onset of sinusoidal microvasculitis. Previous LB to index ones more often displayed the pattern of biliary obstruction in NRH patients without imaging abnormalities in the biliary tree. Biliary features are suspected to be a part of AMR, possibly due to the involvement of peribiliary plexus [15, 29, 36, 37]. Such cases can be speculated as presenting indirect evidence of previous AMR. Previous LB also displayed more rejection. However, features consistent with aAMR were observed in only one NRH patient. Since not all of our patients were biopsied before the index LB, there is the possibility that subclinical “indolent” AMR may have been underdiagnosed during the process. Taken together, we believe that a subset of posttransplant NRH is the result of a form of cAMR with prominent sinusoidal features. This is consistent with the known association of immunological/inflammatory causes of PSVD in native livers and the NRH development [4, 25]. The current Banff criteria for the diagnosis of cAMR in allograft livers require an active/acute component to be present, this definition will miss the cases that have architectural changes and scarring related to previous acute and acute on chronic components, but at the time of biopsy, in particular for protocol biopsies, have no active component. There may be a need to revise the classification to three groups, as has been done in renal transplantation [24], changing the current cAMR to chronic active AMR and then adding a cAMR category where there is no active component, but with documented evidence of previous acute or chronic active AMR.

In conclusion, we reported a subgroup of posttransplant NRH cases (28.5% of the NRH group without a known cause of NRH) with concomitant features consistent with “possible” cAMR according to the current 2016 Banff criteria. The presence of prominent sinusoidal findings led us to suspect the contribution of antibody-mediated sinusoidal injury in the NRH development. Further multicenter studies, with more complete DSA testing, are needed to confirm these findings. To limit costs and potentially pick up AMR at a treatable time point, we recommend that the presence of histological NRH with sinusoidal C4d deposits, especially in the patients without acknowledged etiologies of NRH should prompt DSA testing. The difficulties we have had with classifying cases and the prominent sinusoidal changes warrant a review of the Banff AMR criteria.

## Data Availability

The original contributions presented in the study are included in the article/Supplementary Material, further inquiries can be directed to the corresponding author.

## Supplementary Material

Supplementary Figure S1 Flowchart of the included patients. Patients were initially selected from the Pathology Department database using search criteria prospectively coded for a histological diagnosis of NRH made in 2014. Biopsy-proven NRH patients from this period, with synchronous DSA testing were included. The control group was formed by matching each NRH patient with two non-NRH patients who were biopsied in the nearest timeframe (just before and just after a given NRH patient).

Supplementary Table S1 Histopathological features, immunostaining for C4d and DSA in the NRH group.

## Abbreviations

aAMR, acute antibody-mediated rejection; cAMR, chronic antibody-mediated rejection; DSA, donor specific antibodies; HSC, hepatic stellate cells; LB, liver biopsies; LT, liver transplantation; LFT, liver function tests; LSEC, liver sinusoidal endothelial cells; MFI, mean fluorescence intensity; MHC, major histocompatibility complex; NRH, nodular regenerative hyperplasia; PSVD, porto-sinusoidal vascular disease; TCMR, T cell-mediated rejection; α-SMA, α-smooth muscle actin.
