# Supplementary material for: Evidence for Alloimmune Sinusoidal Injury in De Novo Nodular Regenerative Hyperplasia After Liver Transplantation
Source: Transpl Int. 2023 Jul 26;36:11306. doi: 10.3389/ti.2023.11306 (PMC10409867; doi:10.3389/ti.2023.11306)
Supplement: Supplementary file 1 [file Table1.DOCX]

**Table S1. Histopathological features, immunostaining for C4d and DSA in the NRH group**
